# Altered RBC aggregability in diabetes: a threshold for pathophysiological structure-function RBC changes

**DOI:** 10.1186/s40842-025-00256-2

**Published:** 2025-12-18

**Authors:** Ifechukwude Ebenuwa, Pierre-Christian Violet, Stephanie Teng, Thom Greene, Nicholas Munyan, Razi Berman, Irene Rozga, Mary Walter, Kenneth J. Wilkins, Nermi Parrow, Mark Levine

**Affiliations:** 1 Digestive Diseases Branch, Intramural Research Program, National Institute of Diabetes and Digestive and Kidney Diseases, National Institutes of Health, 10 Center Drive, Bldg. 10 Room 4D51, Bethesda, MD 20892 USA; 2 Clinical Laboratory Core, National Institute of Diabetes and Digestive and Kidney Diseases, National Institutes of Health, Bethesda, MD USA; 3 Biostatistics Program, Office of Clinical Research Support, Office of the Director, National Institute of Diabetes and Digestive and Kidney Diseases, National Institutes of Health, Bethesda, MD USA

**Keywords:** RBC aggregability, Deformability, Osmotic fragility, Diabetes, Vascular complications, RBC structure-function, Hemoglobin-oxygen dissociation

## Abstract

**Objective:**

Vascular complications have been associated with pathophysiological RBC changes including reduced RBC deformability (RBCD): the ability of RBCs to change their shape in the microvasculature and increased red blood cell aggregability (RBCA): the tendency of RBCs to form aggregates (rouleaux). However, understanding of aberrant RBCA in diabetes and its relationship with RBCD is limited. We investigated clinical and RBC structural and functional changes associated with RBCA in comparison with RBCD.

**Methods:**

We conducted an outpatient cross-sectional study of participants with diabetes (*n* = 81) and nondiabetic controls (*n* = 78) at the National Institutes of Health. Clinical history was obtained using standardized forms, fasting blood and urine samples were collected for clinical laboratory measurements and ektacytometry studies of RBC physiological parameters: RBCA, RBCD and osmotic fragility. Functional RBC changes were assessed using hemoglobin-oxygen dissociation (p50). Major outcomes assessed differences in RBCA Aggregation Index (AI), with increased RBCA defined as higher AI. Exploratory outcomes assessed differences in subgroups with type 1 (T1D) and type 2 (T2D) diabetes, and associations with clinical, inflammatory, endocrine and RBC physiological parameters. Outcomes were assessed using both nonparametric and multivariable regression methods.

**Results:**

Compared with controls, AI was significantly higher in the diabetes cohort (75±8 vs 71 ± 11, *p* = 0.007), T2D (77±7 vs 71 ± 11, *p* < 0.001), but not T1D (69±7 vs 71 ± 11, *p* = 0.242). While RBCD values were similar in T1D and T2D (*p* = 0.31), mean AI value was significantly higher in T2D vs T1D (*p* = 0.02). In contrast to RBCD, AI was associated with T2D (*p* < 0.001), BMI (*p* < 0.001), insulin resistance (HOMA-IR, *p* = 0.033), leptin (*p* < 0.001), C-peptide levels (*p* = 0.009), but not vascular complications (*p* = 0.156). While RBCA and RBCD were associated with CRP (*p* < 0.001 and *p* = 0.005 respectively), neither was associated with fibrinogen (*p* = 0.768 and *p* = 0.118 respectively). In contrast to RBCD, AI was associated with increased osmotic fragility (*p* < 0.001) and a threshold of altered hemoglobin-oxygen dissociation (p50) estimated at AI of 75 in both groups using piecewise linear statistical modeling. Thus, AI was positively correlated with p50 at AI < 75 (*p* = 0.033) and negatively correlated at AI ≥ 75 (*p* = 0.007).

**Conclusions:**

Findings show that in contrast to RBCD, RBCA is characterized by a RBC functional threshold beyond which oxygen release is reduced, and RBC structure-function relationship likely modulated by insulin resistance and pro-inflammatory endocrine factors. The distinct but complementary and synergistic changes in RBCD and RBCA provide a framework for strategies aimed at mitigation of vascular risk in diabetes.

**Supplementary information:**

The online version contains supplementary material available at 10.1186/s40842-025-00256-2.

## Introduction

Diabetic vascular complications have been associated with pathophysiological RBC changes including reduced RBC deformability (RBCD): the ability of RBCs to change their shape in the microvasculature and increased red blood cell aggregability (RBCA): the tendency of RBCs to form aggregates, sometimes known as “rouleaux” [1–6]. RBCA and RBCD have been associated with impaired RBC viscosity, microvascular flow, and oxygen-delivering capacity [3–5]. In contrast to RBCD, there is insufficient understanding of the early diabetes-specific changes to RBCA parameters and the associated clinical factors that predispose to vascular complications in populations with diabetes.

With modern ektacytometry, RBCA indices can be accurately and reproducibly quantified using laser-based aggregometry on an ektacytometer platform, obviating past methodological limitations which have resulted in conflicting and contradictory findings [7]. Additionally, modern optical metrics-based ektacytometry offers automated, standardized and reproducible assessment of RBC rheological parameters across physiological and pathological conditions [3]. Using the aggregometer mode of the ektacytometer, a key RBCA measure Aggregation Index (AI) is calculated from static and kinetic parameters (amplitude and half-time respectively) [8]. While ektacytometry has been used to characterize RBCA changes in several chronic diseases and metabolic interventions, no such studies have been conducted in populations with diabetes [9–13].

Additionally, ektacytometry allows standardized measurements of other RBC rheological characteristics including RBCD and osmotic fragility [7, 8]. In a recent study characterizing RBCD in diabetes, aberrant (reduced) RBCD was associated with reduced osmotic fragility and increased hemoglobin-oxygen dissociation, depicting a structure-function relationship [1]. Reduced RBCD was also associated with demographic parameters including Black race, male sex, older age, and clinical parameters including hyperglycemia and vascular complications [1]. However, RBCA is a distinct physiological parameter from RBCD, with reported mechanism linked to inflammatory-sensitive plasma proteins that modulate extrinsic RBC environment with context-dependent effects that may be neutral, anti-aggregating or pro-aggregating [2, 3, 14, 15]. It is therefore reasonable that RBCA could have distinct clinical and physiological differences from RBCD. Characterizing these differences, if they exist, is a necessary step for understanding the diabetes-specific RBC changes that predispose to vascular complications, and strategies aimed at disease prevention and mitigation.

Using AI as our RBCA measure, this study had three objectives. The first was to characterize RBCA differences in diabetes and its variation from RBCD. The second was to investigate associations with diabetes-related clinical, inflammatory and endocrine parameters for both RBCA and RBCD. The third was to investigate structure-function relationships between RBCA and other RBC physiological parameters, including osmotic fragility and hemoglobin-oxygen dissociation (p50).

## Methods

### Study design

This was an outpatient cross-sectional cohort study at the National Institutes of Health Clinical Center and was conducted under protocol 04-DK-0021 (trial registration number NCT00071526) and approved by the Institutional Review Boards of NIDDK, NIH. Inclusion criteria were males and females aged 18-65, with and without type 1 or type 2 diabetes. Exclusion criteria included presence of an acute or chronic illness (other than complications of diabetes mellitus and/or obesity-related conditions) active tobacco use and pregnancy. Participants were recruited from the Washington, DC area using a consecutive sampling approach, with participants recruited and studied on a rolling basis

### Data sources

#### Clinical data

Data were obtained from clinical history, anthropometric measurements and fasting laboratory studies obtained on morning of the single outpatient study visit. Fasting blood and urine chemistry measurements were obtained and measured using standard laboratory methods by the Department of Laboratory Medicine (Clinical Laboratory Improvement Amendments ID number 21D0665373), Clinical Research Center, NIH.

#### Endocrine assays

Enzyme-linked immunosorbent assays (ELISA) were used for measurements of adiponectin (R&D Systems, Minneapolis, MN); leptin (Millipore-Sigma, Burlington, MA); C-peptide and insulin levels (Mercodia, Uppsala, Sweden) and cortisol levels (Assay Genie, Dublin, Ireland).

#### RBC physiology studies

Blood samples for all RBC physiological studies were collected into heparinized tubes (Fisher Scientific) and processed within 4 hours of collection. A standardized sample collection process was used, consistent with the guidelines for hemorheological laboratory techniques [16]. RBCA parameters were measured using the aggregometer mode of the Laser-assisted Optical Rotational Red Cell Analyzer (LORRCA) and conducted per manufacturer’s instruction (R&R Mechatronics, Hoorn, Netherlands). Approximately 1 mL of whole blood was added, and measurements were performed at 37 °C with a camera gain of 208. Following each run, the instrument software produced a syllectogram with the computer-derived RBCA parameters, including a derivative Aggregation Index (AI) [8], such that increased aggregability will be reflected by a comparatively increased AI. AI was selected as the RBCA parameter in this study as AI incorporates both static (amplitude) and kinetic (half-time) parameters [8] and has been used to characterize RBCA differences in clinical studies [9–11].

The RBCD parameter (half maximal shear stress, SS_1/2_) was obtained using shear stress gradient ektacytometry in this cohort, as previously described [1, 8]. RBCs with impaired (reduced) RBCD will have comparatively higher SS_1/2_. For consistency in the comparative analysis, SS_1/2_ was used as a measure of RBCD in this study. Osmotic fragility parameter (minimum osmolality, Omin) was obtained using osmotic gradient ektacytometry as previously described [1, 17], such that increased Omin is indicative of increased osmotic fragility, while decreased Omin is indicative of reduced osmotic fragility. Hemoglobin-oxygen dissociation (p50) was assessed using a HEMOX Analyzer (TCS Scientific Co., New Hope, PA) as previously reported [1, 18]. Higher p50 values indicate increased hemoglobin-oxygen dissociation, while lower p50 values indicate decreased hemoglobin-oxygen dissociation.

### Study outcomes

The primary outcome assessed differences in RBCA measures between participants with and without diabetes. Exploratory outcomes assessed associations with clinical parameters and RBC physiological measures: RBCD (SS_1/2_), osmotic fragility (Omin), and hemoglobin-oxygen dissociation (p50).

### Statistical methods

Descriptive statistics were calculated for baseline characteristics of study participants. Counts and proportions were used to describe categorical variables and associated comparisons using either Pearson’s chi-square test or Fisher’s exact test as indicated by expected cell counts. Mean and standard deviations (or median and interquartile range) were used to describe continuous variables, and associated comparisons used Welch’s t-test or Wilcoxon-Mann-Whitney tests, as indicated by assumptions. The primary outcome characterizing differences in RBCA measures across diabetes status groups was assessed using Wilcoxon-Mann-Whitney tests, leveraging the Hodges-Lehmann estimator for shift in distribution between the pair of groups’ respective values. To account for group differences in baseline demographic covariates, change-of-estimate analysis using linear regression models assessed confounders for RBCA parameters.

RBCA measures, stratified by diabetes status were transformed using Box-Cox transformations. All models report estimated regression coefficients, associated 95% confidence intervals, and p-values. Given the exploratory intent of these analyses, multiplicity adjustments were not reported. To assess relationships with other RBC physiological parameters, bivariate analyses were conducted using scatterplots and Pearson correlation coefficients to assess associations between RBCD and RBCA measures, stratified by diabetes status. Tertiles for diabetes-status-specific AI were calculated and relationships with other RBC physiology measures were investigated using linear regression. Thresholds for the association between AI and hemoglobin-oxygen dissociation were developed using a piecewise linear model of the association for pooled cohorts as follows: enrollment-cohort stratification; Box–Cox transformation; exploratory tertiles for visualization; piecewise linear modeling with maximum likelihood estimation for the breakpoint and bootstrap confidence intervals. AI tertiles were for exploration/plotting, whereas the threshold was defined as the changepoint in association and derived from the continuous model. Refer to Supplementary Methods for more details.

All analyses assumed 5% significance, with missing values handled using available- or complete-case analyses, as applicable per use of repeated measures data. Analyses were conducted using SAS v9.4 (PROC GENMOD) and R version 4.0 or higher with packages car, chest, DescTools, forestplot, geepack, ggplot2, glmnet, haven, MASS, SiZer, tidyverse.

## Results

### Enrollment flow and baseline characteristics of participants with and without diabetes

Among the individuals assessed for eligibility, 81 participants with diabetes (Type 1 and Type 2) and 78 without diabetes (nondiabetic controls) were included in final analysis (Table 1, Supplementary Figure 1A). In the primary comparator groups, race/ethnic and sex composition were not significantly different, however the mean age was significantly higher in the diabetes cohort vs nondiabetic controls (49±12 vs 34 ± 13 years, *p* < 0.05, Table 1). Among the baseline clinical parameters, the diabetes cohort had higher BMI (33±7 vs 27 ± 5 Kg/m^2^, *p* < 0.05), fasting glucose (146±53 vs 88 ± 9 mg/dL, *p* < 0.05), hemoglobin A1c (7.9±1.5 vs 5.4 ± 0.4%, *p* < 0.05), protein-to-creatinine ratio [PCR](0.270 vs 0.096 mg/mg, *p* < 0.05) and lower eGFR (101±26 vs 113 ± 21 ml/min/1.73 m^2^, *p* < 0.05), Table 1.

**Table 1:**
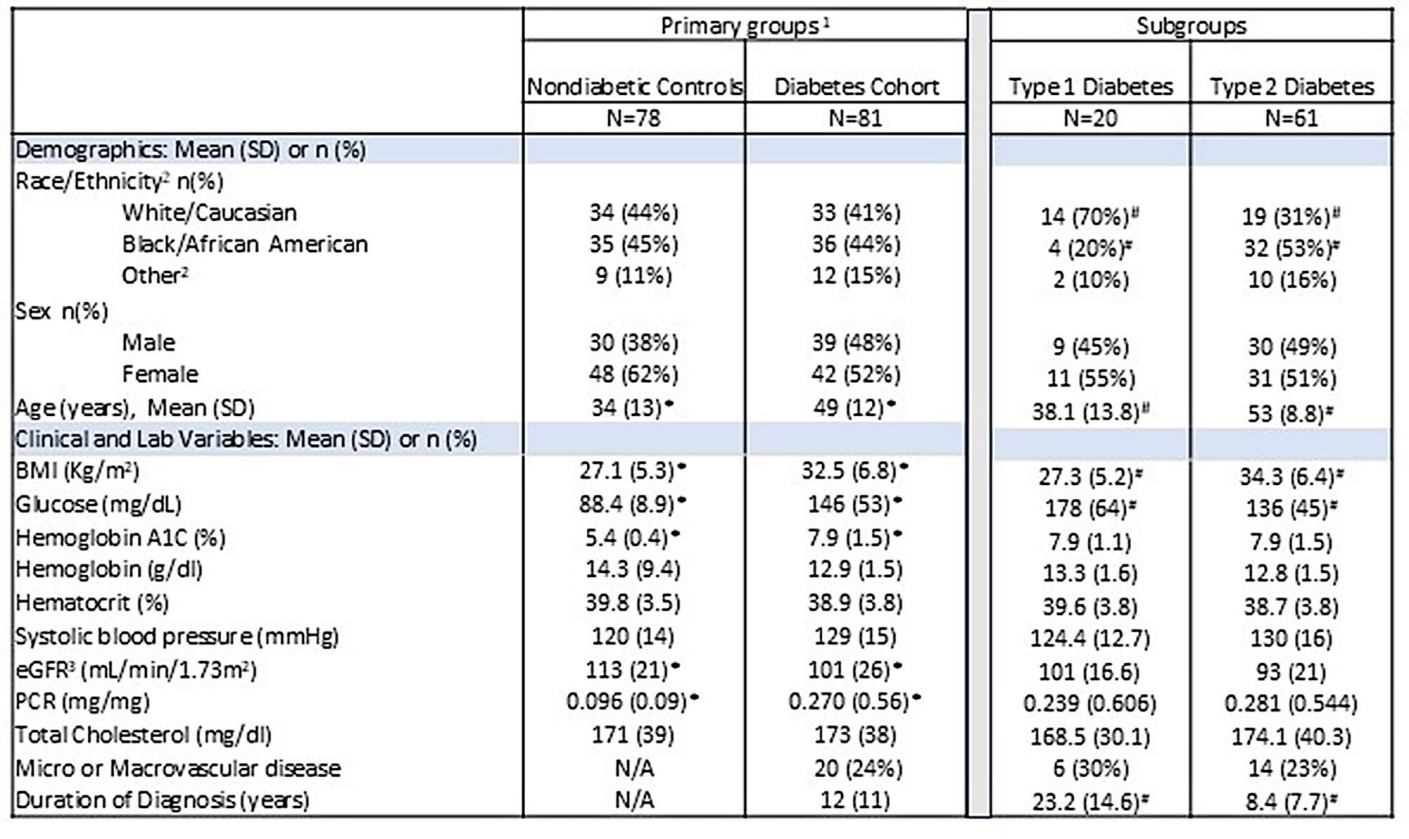
Baseline demographic and clinical characteristics of participants in the diabetes cohort (*N* = 81), nondiabetic controls (*N* = 78) and subgroups with type 2 (*N* = 61) and type 1 (*N* = 20) diabetes

^1^Enrollment-based groups based on study design

Among participants in the diabetes cohort (*N* = 81), 75% (*N* = 61) had type 2 and 25% (*N* = 20) type 1 diabetes; 24% had micro or macrovascular complications (diabetic retinopathy, neuropathy, nephropathy, peripheral vascular disease), mean duration of diabetes was 12 years (Table 1). Compared to the subgroup with type 1 diabetes, the subgroup with type 2 diabetes had a lower proportion of Black/African Americans (53 vs 20%), similar proportion of females (51 vs 55%), older age (53 vs 38 years), higher BMI (34 vs 27 Kg/m^2^), lower fasting glucose (136 vs 178 mg/dL) but similar hemoglobin A1c (7.9 vs 7.9%), Table 1.

### Differences in RBCA measures in participants with vs without diabetes

**Fig. 1:**
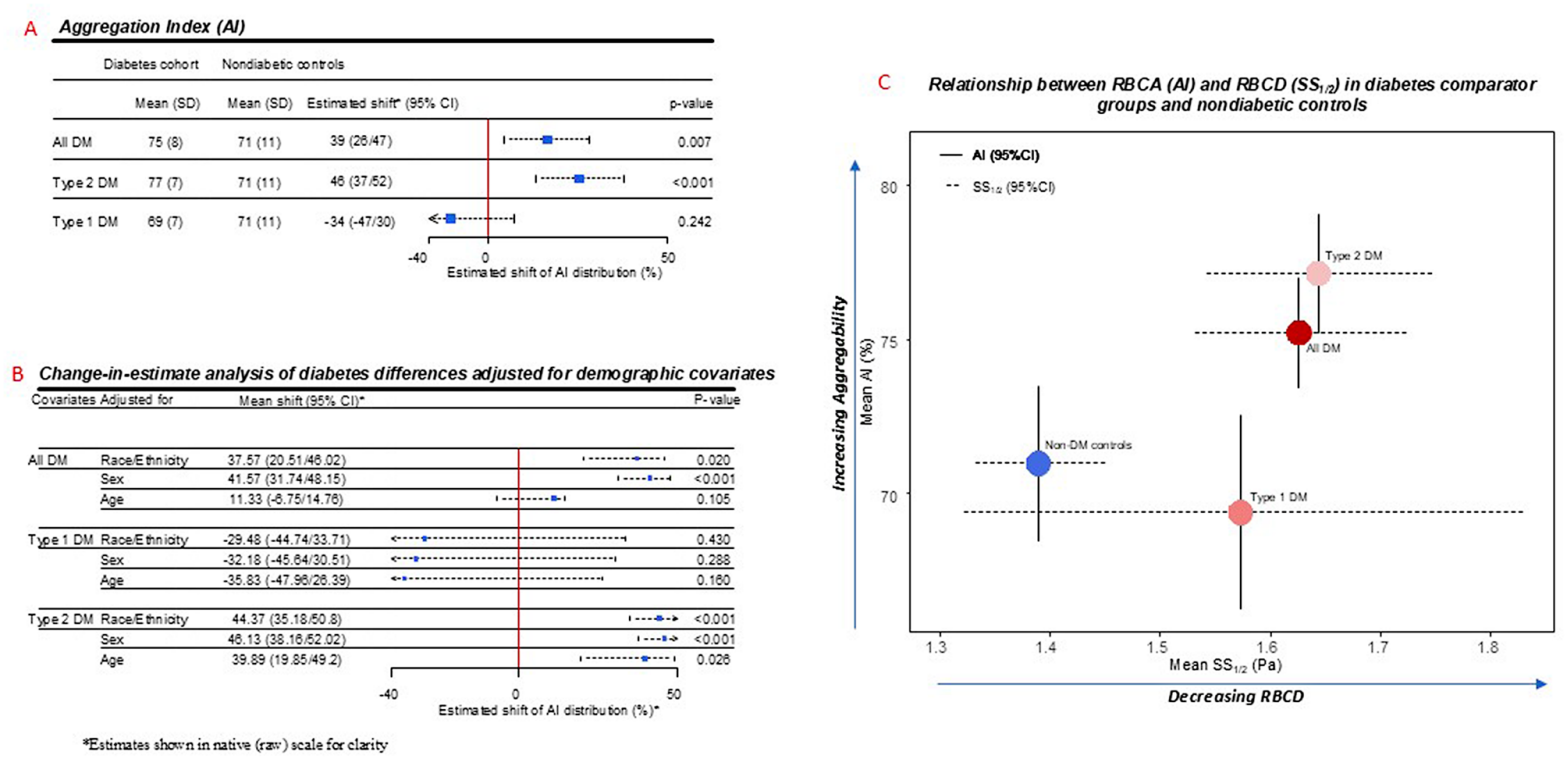
RBCA (AI) differences in diabetes comparator groups, and relationship with RBCD. (**A**) Mean AI values and estimated shifts in distribution on the Box-Cox transformed scale across comparator groups. Squares indicate Hodges-Lehmann shifts, with horizontal dotted lines indicating 95% confidence interval (CI). The CI margins show either a vertical bar symbol reflecting actual values or an arrow symbol indicating visual margins were abbreviated for visual clarity. For clarity, values are shown in native (untransformed) scale. (**B**) Change-in-estimate analysis of diabetes differences adjusted for demographic covariates. Squares indicate Hodges-Lehmann shifts, with horizontal dotted lines indicating 95% confidence interval (CI). The CI margins show either a vertical bar symbol reflecting actual values or an arrow symbol indicating visual margins were abbreviated for visual clarity. (**C**) Relationship between mean RBCA (AI) and RBCD (SS1/2) in diabetes comparator groups and nondiabetic controls. Horizontal lines indicate 95% confidence interval for RBCD (SS1/2), and vertical lines indicate 95% confidence interval for RBCA (AI). Abbreviations: AI, Aggregation Index; DM, diabetes mellitus; nDM, non-diabetic controls

Compared with nondiabetic controls, the Aggregation Index (AI) was significantly higher in the diabetes cohort (75±8 vs 71 ± 11, *p* = 0.007), with even higher AI in the subgroup with type 2 diabetes (77±7 vs 71 ± 11, *p* < 0.001), while the subgroup with type 1 diabetes was not significantly different from controls (69±7 vs 71 ± 11, *p* = 0.242), Fig. 1A.

Change-in-estimate analysis with adjustments for differences in demographic parameters were conducted among the diabetes comparator groups relative to controls (Fig. 1B). Relative to controls, AI differences persisted in the diabetes cohort and subgroup with type 2 diabetes following adjustments for race/ethnicity (*p* = 0.02 and *p* < 0.001 respectively) and sex (*p* < 0.001 and *p* < 0.001 respectively), Fig. 1B. However, following adjustment for age differences, only the subgroup with type 2 diabetes remained significantly different from controls (*p* = 0.026). Lack of significant difference in the subgroup with type 1 diabetes vs nondiabetic controls was unchanged following adjustments for race/ethnicity, sex and age (*p* = 0.43, *p* = 0.288 and *p* = 0.160 respectively), Fig. 1B.

### Differences in RBCA (AI) and RBCD (SS_1/2_) among diabetes subgroups with type 1 and type 2 diabetes

We compared differences in AI and RBCD (SS_1/2_) between diabetes subgroups (type 1 vs type 2 diabetes), Fig. 1C. Along the vertical axis (AI), mean AI values were significant different between the diabetes subgroups (*p* = 0.02), with the largest mean AI values in the subgroup with type 2, and the smallest mean AI values in the subgroup with type 1, which also demonstrated mean AI values below the nondiabetic controls (Fig. 1C). In contrast to AI, along the horizonal axis, mean RBCD values in the diabetes subgroups were not different (*p* = 0.31, Fig. 1C).

### Association between AI, demographics and clinical parameters

**Fig. 2:**
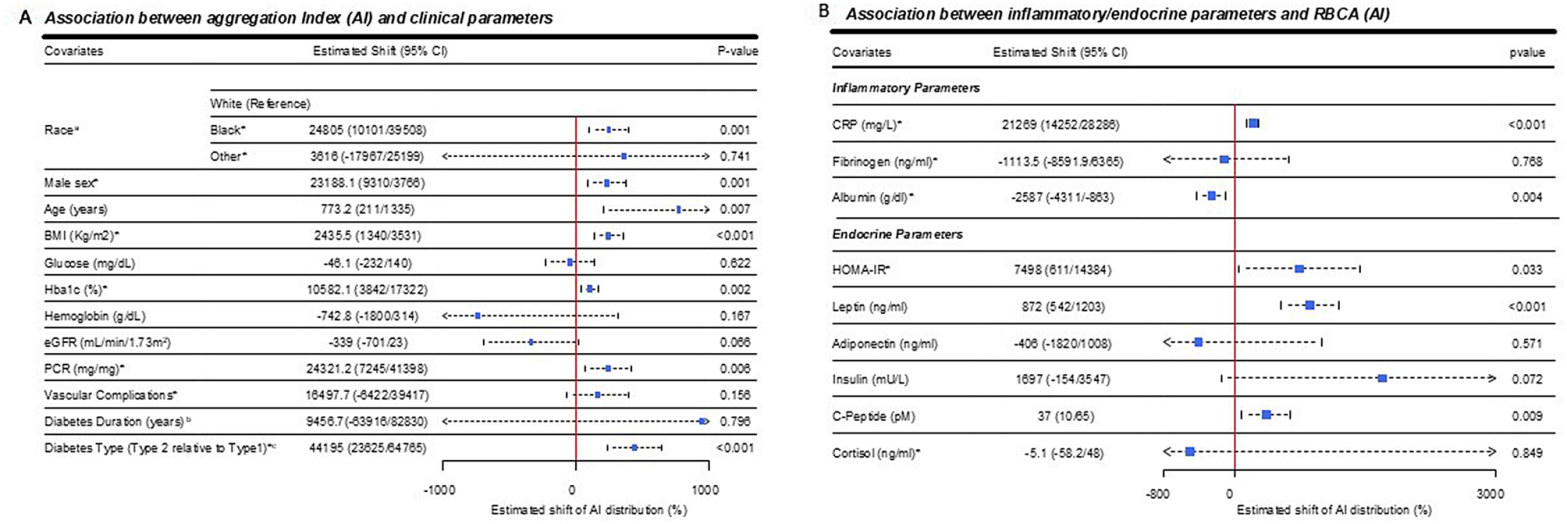
Associations between RBCA (AI), demographics, clinical, inflammatory and endocrine parameters using diabetes-status-adjusted linear regression on Box-Cox transformed scale for AI in all participants. Association between AI and clinical variables (**A**), inflammatory and endocrine parameters (**B**). Squares indicate regression-modeled shifts in mean AI on the Box-Cox transformed scale, with horizontal dotted lines indicating 95% CI. The CI margins show either a vertical bar symbol reflecting actual values or an arrow symbol indicating visual margins were abbreviated for visual clarity. ^a^ Black and White groups were comprised of non-Hispanic participants. “Other” was comprised of Asians, Hispanic/Latino ethnicity, and multiracial participants. ^b^ Presence of micro- or macrovascular complications. ^c^ Type 2 relative to type 1 diabetes. *values were adjusted (divided or multiplied by 10 or 100) to fit the scale of the forest plot. Abbreviations: AI, Aggregation Index; BMI, body mass index; eGFR, estimated glomerular filtration rate; PCR, protein-to-creatinine ratio; HOMA-IR, homeostatic model assessment for insulin resistance

As with previously reported clinical associations with RBCD [1], increased AI was significantly associated with Black race [relative to White race] (*p* = 0.001), male sex (*p* = 0.001), older age (*p* = 0.007), hemoglobin A1c (*p* = 0.002) and protein-to-creatinine ratio (*p* = 0.006), Fig. 2A. In contrast to RBCD however, AI was associated with higher BMI (*p* < 0.001) and type 2 diabetes [relative to type 1] (*p* < 0.001). Also, in contrast to RBCD, AI was not associated with fasting glucose concentration, hemoglobin concentrations, or vascular complications (*p* > 0.05 for all) Fig. 2A.

### Association between inflammatory/endocrine parameters and RBCA (AI)

Based on reported mechanisms with inflammatory-sensitive proteins [6, 19, 20] and significant association with diabetes types (Figs. 1C and 2A), we investigated associated inflammatory and endocrine parameters linked to either inflammation or differences in diabetes type. Among the inflammatory markers, CRP was associated with increased AI (*p* < 0.001), albumin values were associated with reduced AI (*p* = 0.004) and surprisingly, fibrinogen was not associated with AI (*p* = 0.768), Fig. 2B. Among the endocrine parameters, increased AI was significantly associated with Homeostatic Model Assessment for Insulin Resistance [HOMA-IR] (*p* = 0.033), higher leptin (*p* < 0.001) and C-peptide concentrations (*p* = 0.009), but not adiponectin, insulin or cortisol levels (*p* > 0.05 for all), Fig. 2B. To determine what associated parameters were specific to AI, we analyzed similar associations with RBCD (Supplementary Figure 2). In contrast to AI, reduced RBCD (higher SS_1/2_) was associated with plasma cortisol (*p* < 0.001) but not HOMA-IR (*p* = 0.198), leptin (*p* = 0.055) or C-peptide (*p* = 0.822), Supplementary Figure 2.

To further explore the clinical, inflammatory and endocrine associations with AI within study cohorts, we assessed associations in subgroups stratified by AI tertiles (Table 2). Among subgroups in the diabetes cohort, compared with the low-tertile subgroup, high-tertile subgroup had significantly higher proportion of type 2 diabetes (93 vs 57%, *p* = 0.028), lower proportion of type 1 diabetes (7 vs 43%, *p* = 0.028), higher CRP (*p* = 0.003), HOMA-IR (*p* = 0.002), insulin concentrations (*p* = 0.003), C-peptide levels (*p* = 0.002), leptin (*p* = 0.007), adiponectin (*p* < 0.001), however cortisol values were not different (*p* = 0.922), Table 2. A similar pattern was observed among the nondiabetic controls, with comparatively higher CRP (*p* < 0.001), HOMA-IR (*p* = 0.004), Insulin (*p* = 0.003), C-peptide (*p* = 0.01), leptin (*p* = 0.001), and cortisol (*p* = 0.043), however adiponectin was not different (*p* = 0.556), Table 2.

**Table 2:**
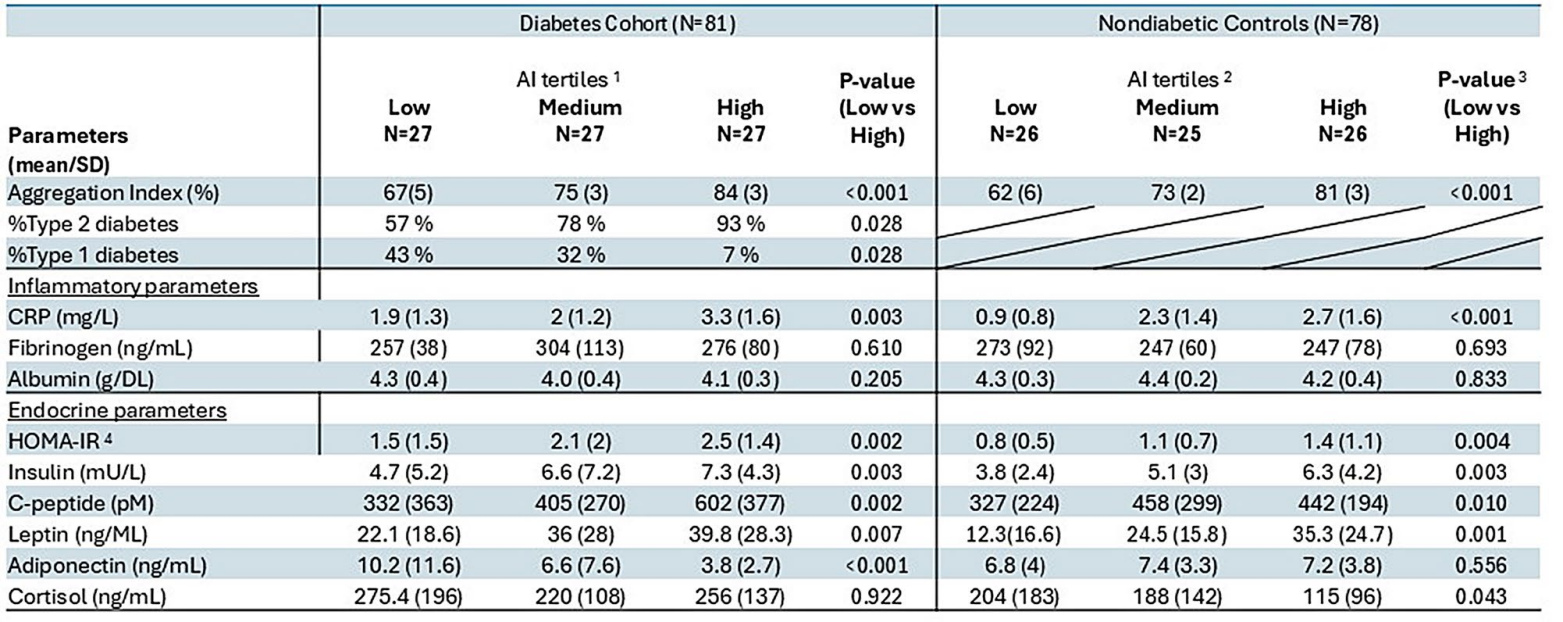
Mean values for inflammatory/endocrine parameters in diabetes and control subgroups stratified by AI tertiles

^1^AI tertile ranges for the diabetes cohort: low tertile (52.1–71.6); medium tertile (71.7–80.1), high tertile (80.2–93)

### AI threshold and RBC functional parameter: hemoglobin-oxygen dissociation (p50)

**Fig. 3:**
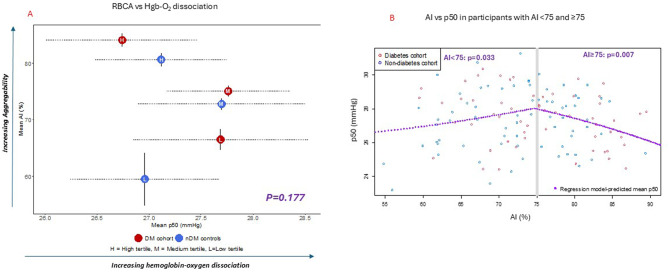
Relationship between RBCA and hemoglobin-oxygen dissociation (p50) in the diabetes cohort (*N* = 58) and nondiabetic controls (*N* = 75). (**A**) Association between mean RBCA (AI) and mean p50 values in subgroups stratified by ai tertiles within the diabetes cohort (red circles) and nondiabetic controls (blue circles); horizontal dotted lines indicate 95% confidence interval for p50, and vertical lines indicate 95% confidence interval for RBCA (AI). (**B**) unstratified AI-p50 associations in all participants, back-transformed to the native scales to exhibit statistically significant and qualitatively distinct associations above and below the estimated ai threshold of ≈75. Note that, to meet assumptions inherent to estimating threshold via piecewise regression, we report the distinct positive-then-negative association measures from either side of the threshold as maximum-likelihood estimated slopes linear on the Box-Cox scale (see supplemental methods for more detail). Abbreviations: AI, Aggregation Index; DM, diabetes mellitus; nDM, non-diabetic controls; L: low AI tertile, M: medium AI tertile, H: high AI tertile

We assessed the association between RBCA and p50 in subgroups stratified by AI tertiles (Fig. 3, Table 2, Supplementary Table 1). Diabetes and control subgroups were interspersed with each other to form a horizontal V-shaped curve, with an inflection point suggestive of an AI threshold—a range of AI values in which AI-p50 association changes directionality from positive to negative (Fig. 3A). Using mathematical formulation of piecewise-linear modeling that allows for multiple changepoints along the range of observed values, we estimated a single changepoint indicating this AI threshold range to be spanning a value of approximately 75 in both diabetes and control groups, bootstrap-based 95% confidence interval 61.8–81.0 (see Supplementary Methods). This V-shaped pattern and observed threshold with RBCA and p50, was in marked contrast to the constant linear positive association observed with RBCD (Supplementary Figure 3A) [1].

While there was no association between AI and p50 across all subgroups (*p* = 0.177, Fig. 3A), significant differences emerged when all participants were stratified based on AI values below or above the estimated AI threshold of 75; AI exhibited a statistically significant positive association with p50 below this threshold (Corr.0.25, CI: 0.02 to 0.45, *p* = 0.0336), and a statistically significant negative correlation with p50 above this threshold (Corr. −0.35, CI:-0.55 to −0.10, *p* = 0.007), Fig. 3B. This statistically significant and qualitatively divergent set of association conclusions, relative to the estimated changepoint, confirmed the possible presence of an AI-based threshold in relation to RBC function (p50).

### Association between RBCA (AI) and structural RBC measures: osmotic fragility & RBCD (SS_1/2_)

**Fig. 4:**
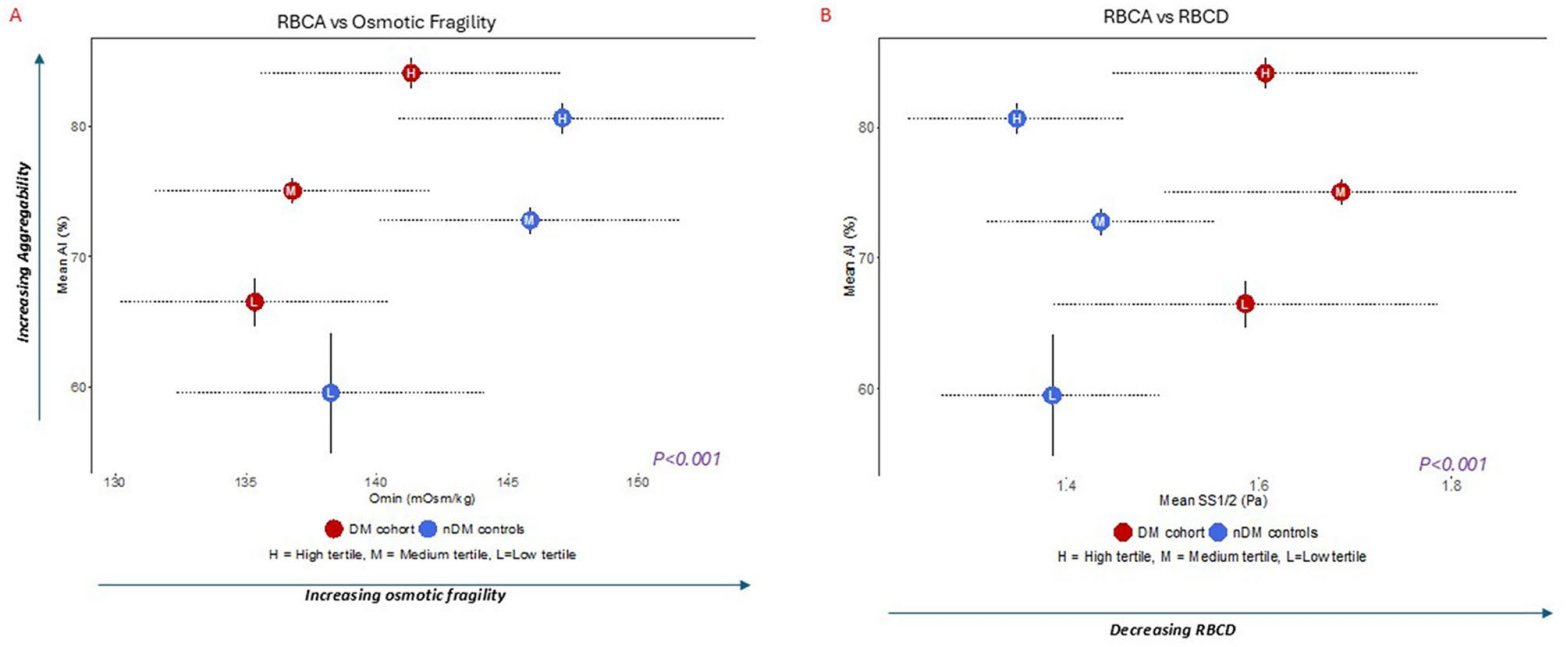
Relationships between RBCA, osmotic fragility (Omin) and RBCD (SS1/2) in the diabetes cohort and nondiabetic controls. Association between mean RBCA (AI), mean osmotic fragility values stratified by (**A**) and RBCD values (**B**) in subgroups stratified by AI tertiles within the diabetes cohort (red circles) and nondiabetic controls (blue circles); horizontal dotted lines indicate 95% confidence interval for Omin or SS1/2, and vertical lines indicate 95% confidence interval for RBCA (AI). Abbreviations: AI, Aggregation Index; DM, diabetes mellitus; nDM, non-diabetic controls; RBCA, Red Blood Cell Aggregability; RBCD, Red Blood Cell Deformability

We investigated associations between RBCA and osmotic fragility—a structural RBC physiological parameter, in subgroups stratified by AI tertiles (Fig. 4A, Supplementary Table 1). Mean osmotic fragility values for the combined diabetes groups were distinctly lower than for the controls (Fig. 4A), consistent with our previous description of reduced osmotic fragility in diabetes. [1] In all subgroups combined, AI was significantly associated with increased osmotic fragility (*p* < 0.001 all, Fig. 4A). This positive association between osmotic fragility and AI contrasted with previously reported negative association with RBCD (Supplementary Figure 3B) [1]. While this study was underpowered to assess differences in AI-osmotic fragility association below and above the threshold, the slope seven-fold higher above the threshold (Slope 7.5e-07, CI: −8.0e-07 to 2.3e-06) vs below the threshold (Slope 1.1e-07, CI: −1.3e-06 to 1.5e-06), Supplementary Figure 4A. Findings indicated comparatively stronger association above vs below the threshold.

Mean RBCD values in the combined diabetes groups stratified by AI tertiles were distinctly larger the controls, indicating reduced RBCD in diabetes (higher SS_1/2_, Fig. 4B) [1]. Overall association between AI and RBCD across all subgroups was significant (*p* < 0.0001, Fig. 4B). AI-RBCD slope was more than ten-fold larger below the threshold (Slope 2.4e-06, CI: −5.4e-07 to 5.3e-06) vs above the threshold (Slope: 2.4e-12, CI: −3.0e-06 to 3.2e-06), Supplementary Figure 4B. Findings indicated that in contrast to osmotic fragility, AI-RBCD association was comparatively stronger below vs above the threshold.

## Discussion

In this study, we investigated RBCA (AI) changes in diabetes and characterized differences with RBCD using standardized ektacytometry measurements in similar cohorts [1]. In contrast to RBCD, increased AI was significantly associated with type 2 diabetes and insulin resistance, pro-inflammatory endocrine hormones and most notably, the presence of an AI threshold: An inflection point in which increased AI values became associated with decreased hemoglobin-oxygen dissociation (p50), a functional RBC measure. Using piecewise linear statistical modeling, the estimated AI threshold was approximately 75, with similar estimates in both diabetes and control groups (Supplementary Methods). While threshold patterns were similar in both diabetes and control groups, the pooled data was used to optimize statistical power in assessing dichotomized AI-p50 correlations below and above this threshold, as each individual cohort alone was underpowered to establish non-null association on both sides of any putative threshold. Thus, increased AI was positively correlated with p50 below this threshold (*p* = 0.03) and negatively correlated with p50 above this threshold (*p* = 0.007, Fig. 3). In this first characterization of RBCA in diabetes using modern ektacytometry techniques, findings suggest RBCA is associated with an AI threshold of altered RBC pathophysiological changes in diabetes.

**Fig. 5:**
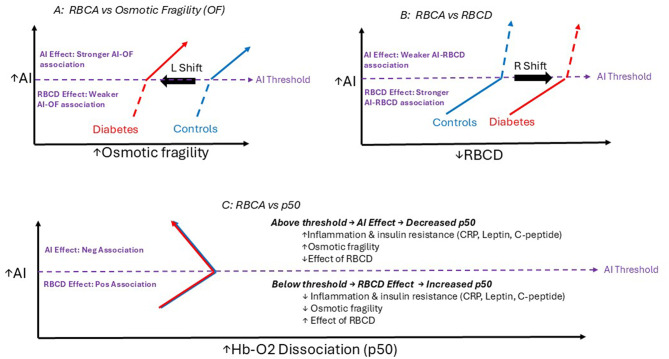
Scheme illustrating structure-function relationships between RBCA and RBC parameters in diabetes

Our data also showed that RBCA was associated with structural RBC parameters: osmotic fragility and RBCD. Analysis of these measures were essential in characterizing the RBCA structure-function relationships and possible mechanistic basis for an AI threshold. Based on our data, two concurrent and complementary rheological processes may account for these structure-function changes: “RBCD effect” and “AI effect” (Fig. 5). The “RBCD effect” are diabetes-specific changes mechanistically linked to RBC dehydration in diabetes [1, 17, 21], including (1) decreased osmotic fragility in diabetes relative to controls as reflected by the “left shift” in Fig. 5A, (2) decreased RBCD in diabetes relative to controls as reflected by right shift in Fig. 5B, (3) compensatory increase in hemoglobin-oxygen dissociation (p50) with incremental decrease in RBCD as previously described in RBCD-stratified subgroups (Supplementary Figure 3A) [1], and (4) Comparatively stronger AI-RBCD slopes below vs above the threshold (Supplementary Figure 4B). Taken together, findings indicate that at lower AI values below the threshold, RBCD effect is the predominant process that likely accounts for the positive association with increased p50 (Fig. 5C) [1].

The 2^nd^ rheological process, “AI effect”, reflects intrinsic and extrinsic (plasma) RBC factors uniquely associated with AI, but not RBCD. The most notable intrinsic factor was the positive association with osmotic fragility (*p* < 0.001, Fig. 4A), reflected by incrementally higher osmotic fragility values from low-to-high AI-tertile subgroups (Supplementary Table 1). Above the AI threshold, the “AI effect” is predominant, as indicated by higher AI and osmotic fragility values (Figs. 4 and 5) and comparatively stronger AI-osmotic fragility slopes above vs below the threshold (Supplementary Figure 4A). As previously reported, osmotic fragility is associated with decreased p50 [1], a possible explanation for the negative AI-p50 association above the threshold (Figs. 3 and 5C). Findings indicate that beyond an RBCA threshold, oxygen release is reduced, perhaps as a decompensatory response. Despite higher osmotic fragility values in high- vs low-AI tertiles within each group, osmotic fragility values in all diabetes subgroups were lower than controls (“left shift” in Figs. 4A and 5A) suggesting the mechanistic basis for “AI effect” are likely based on the modulating effect of extrinsic plasma factors (Table 2) rather than cytoskeletal changes or membrane dehydration [6, 15, 22, 23]. Given our relatively well controlled cohorts, it is possible cohort studies with higher inflammatory and vascular disease burdens would yield a more dominant “AI effect” with RBC cytoskeletal changes more typical of enhanced osmotic fragility and propensity for hemolysis [17].

Based on previous studies, a key mechanistic difference between RBCD and RBCA is the role of extrinsic RBC factors (“plasma factors” or “inflammatory sensitive proteins”) that alter the biochemistry of the RBC membrane and predispose to increased RBCA [6, 22, 23]. Given our limited understanding of RBCA changes in diabetes, a first step was to investigate clinical parameters exclusively associated with RBCA (and not RBCD), as a pre-requisite for identifying possible plasma factors in our study cohorts. In contrast to RBCD, AI was associated with type 2 diabetes (Figs. 1 and 2). Among the high-tertile diabetes subgroup, 93% had type 2 in comparison to 7% with type 1 (Table 2). And despite the comparatively smaller number of participants with type 2 diabetes (*N* = 61) vs total diabetes cohort (*N* = 81) [Table 1], only the subgroup with type 2 diabetes maintained the significant difference in AI following adjustment for age differences (Fig. 1B). These data suggests that difference in the combined diabetes group was attenuated by participants with type 1 diabetes as subsequent adjustments for demographic differences yielded comparatively lower AI estimates in type 1 (albeit statistically insignificant), in contrast to higher AI estimates in subgroup with type 2 diabetes (Fig. 1).

Based on our data, the most likely explanation for discordant patterns in type 1 vs type 2 diabetes are the metabolic and endocrine parameters specifically associated with AI—but not RBCD, including BMI, HOMA-IR, C-peptide and leptin values (Fig. 3, Supplementary Fig. 2). These parameters are more commonly associated with insulin resistance phenotype of type 2 diabetes, in contrast with the insulin deficient phenotype of type 1 diabetes [24]. Another possible explanation is reduced sample size of type 1, however based on trends in change-in-estimate analyses (Fig. 1B), it is much more plausible that expanded sample size of participants with type 1 would result in lower—not higher AI values relative to controls. Additionally, given similar hemoglobin A1c values (Table 1), glycemic patterns would not account for these observations.

While HOMA-IR—a derivative of fasting glucose and insulin—was associated with increased AI, fasting glucose and insulin concentrations were not, suggesting dysregulation in proportion of insulin-to-glucose ratio as an underlying factor in increased AI. Additionally, given that both C-peptide and insulin levels reflect endogenous insulin status, the significant association with C-peptide–but not insulin levels, raises the possibility that excess C-peptide exerts pathophysiological changes in RBCs that is independent of endogenous insulin production. While several studies have investigated the effects of insulin and C-peptide on RBC physiological parameters, precise mechanisms related to RBCA are unknown [25–29]. Findings highlight our limited understanding of how endocrine changes disrupts the structure and function of RBCs.

It was notable that among the nonspecific inflammatory parameters, RBCA was negatively associated with albumin (*p* = 0.004) but surprisingly, RBCA was not associated with fibrinogen (*p* = 0.768, Fig. 2), which is inconsistent with previous studies describing fibrinogen as a key plasma factor in increased RBCA [6, 30]. This may be explained by three possible factors. First, in contrast with most studies that observed significant association between fibrinogen and increased RBCA, our cohort did not include participants with ischemic heart disease or coronary artery disease (CAD) which are associated with highly elevated fibrinogen levels [6, 15, 31–34]. Our cohort included relatively well controlled participants with diabetes, thus lower fibrinogen levels. Second, is the possible threshold effect of fibrinogen, such that pro-aggregating properties occurs above a threshold plasma fibrinogen concentrations > 450 mg/dL [15], well above the values observed in our cohorts (Table 2). Third, is the context-dependent nature in which plasma proteins may exert neutral, pro- or anti-aggregating effects, based on concentrations of related plasma proteins including albumin and immunoglobulin levels [14, 15]. While immunoglobulin levels were not obtained in this study, our data showing significant anti-aggregating effect of albumin (Fig. 2B) in the context of low fibrinogen levels is consistent with this explanation, while pro-aggregating effect of albumin would be expected in a population with higher fibrinogen levels [14, 15].

Additionally, while RBCA was significantly associated with higher CRP (*p* < 0.001), this association was not exclusive to RBCA, as CRP was also associated with reduced RBCD (*p* = 0.005, Supplementary Fig. 2). Taken together, findings suggest that in our study cohorts, the strongest associations with extrinsic “plasma factors” specific to RBCA and not RBCD, were insulin resistance and pro-inflammatory endocrine measures, rather than non-specific inflammatory proteins such as CRP. One possible explanation for these observations is the RBCA threshold concept, such that a much higher number and proportion of participants above the RBCA threshold are needed to observe discernible associations with parameters such as fibrinogen, in contrast with insulin resistance and pro-inflammatory endocrine parameters, as observed in our generally healthy and well controlled cohorts. Beyond endocrine and inflammatory factors, RBC aging/senescence has also been associated with increased RBCA and decreased RBCD, with potential mechanisms that include membrane glycation/oxidation, sialic-acid loss/glycocalyx alterations, eruptions in nitric oxide signaling among other possibilities [35, 36].

The RBCA threshold concept may also explain differences in clinical associations between RBCD and RBCA. Among the glycemic parameters, AI was associated with increased HgbA1c, but not fasting glucose values (Fig. 2), in contrast to RBCD that was associated with both fasting glucose and HgbA1c.^1^ These data suggest that in contrast to RBCD, AI is specifically associated with chronic hyperglycemia—with sufficient duration of glycemia rather than transient glycemic changes, consistent with studies showing linking chronic hyperglycemia with pathophysiological RBC membrane changes [37, 38]. Similarly, in contrast to RBCD, RBCA was not associated with vascular complications (Fig. 2) [1], likely due to the higher threshold of disease pathology and pathophysiological changes (e.g. higher fibrinogen levels) needed to discern changes On this basis, presence of a threshold effect in RBCA, but not RBCD, makes RBCD a more sensitive indicator of early vascular complications in healthier cohorts, as a comparatively larger proportion of participants above the RBCA threshold may be needed to effectively investigate associations between RBCA and vascular complication. Conversely, RBCA might be a more sensitive marker of pathophysiological changes in cohorts with advanced disease burden (e.g. CAD), or in tracking responses to modifiable risk factors targeted at mitigating insulin resistance, such as lifestyle changes and medications.

This study had several strengths. This is the first comprehensive analyses investigating RBCA changes in diabetes using modern ektacytometry measurements with diabetes-focused outcome measures. The standardized and automatized ektacytometry in similar cohorts allowed comparisons of RBC physiologic parameters (RBCA, RBCD, Osmotic fragility) with improved accuracy and reproducibility—essential in uncovering an RBCA threshold, when coupled with functional outcome measure (p50). Additionally, this study includes a comprehensive analysis of clinical, inflammatory and endocrine associations pertinent to a diabetes population. This study also had several limitations. Because recruitment was based on diabetes diagnosis—not diabetes type, there was no a priori strategy to have balanced proportions of participants with type 1 and type 2 diabetes. Additionally, while the cross-sectional study design is a necessary first step in understanding clinical relationships and associations, causality cannot be established.

In conclusion, this study showed that RBCD and RBCA have distinct but complementary RBC structure-function relationships in diabetes, and associated with distinct clinical, inflammatory and endocrine factors. Larger and longitudinal studies are needed to better understand mechanisms and clinical implications.

## Electronic supplementary material

Below is the link to the electronic supplementary material.

Supplementary Material 1

Supplementary Material 2

## Data Availability

Data described in the manuscript will be made available upon request pending application and approval.

## Abbreviations

AI: Aggregation Index
AMP: Amplitude
BMI: Body mass index
eGFR: Estimated glomerular filtration rate.
CAD: Coronary artery disease
HbA1c: Hemoglobin A1c
p50: Hemoglobin-oxygen dissociation
HOMA-IR: Homeostatic Model Assessment of Insulin Resistance
LORRCA: Laser-assisted Optical Rotational Red Cell Analyzer
Omin: Minimum osmolality
PCR: protein creatinine ratio.
RBC: Red blood cell
RBCA: Red blood cell aggregability
RBCD: Red blood cell deformability
SS: Shear stress
SS_1/2_: Half-maximal shear stress
T_1/2_: Aggregation half-time
T1D: Type 1 diabetes
T2D: Type 2 diabetes
