# Supplementary material for: Altered RBC aggregability in diabetes: a threshold for pathophysiological structure-function RBC changes
Source: Cardiovasc Diabetol Endocrinol Rep. 2025 Dec 18;11:44. doi: 10.1186/s40842-025-00256-2 (PMC12713270; doi:10.1186/s40842-025-00256-2)
Supplement: Supplementary file 1 — Supplementary Material 1 [file 40842_2025_256_MOESM1_ESM.pdf]

Supplementary Figure 1

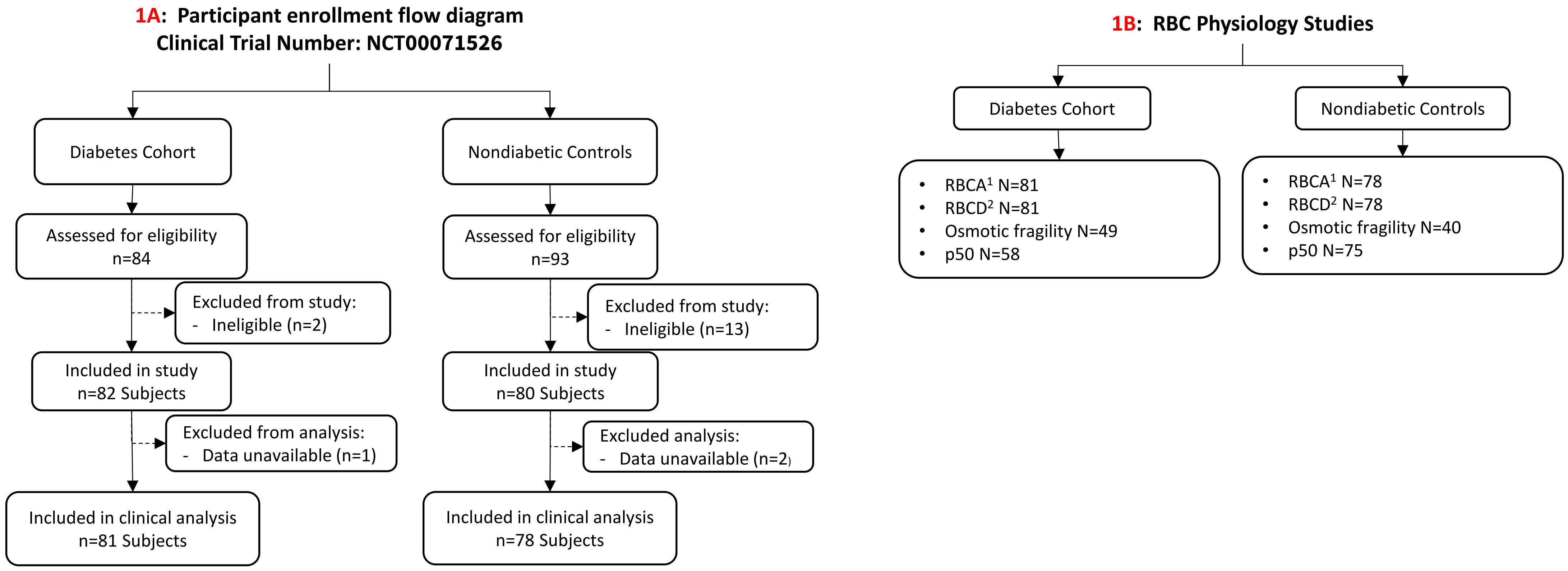

(A) Enrollment and patient flow of study participants in the diabetes cohort and nondiabetic control group, (B) Flow diagram for RBC physiology studies conducted in samples obtained from participants both groups.

[1] RBCA: Red blood cell aggregability

[2] RBCD: Red blood cell deformability

Supplementary Figure 2: RBCD ($SS_{1/2}$): Endocrine & Inflammatory parameters

Association between inflammatory/endocrine parameters and RBCD ($SS_{1/2}$)

Covariates	Estimated Shift (95% CI)	pvalue
Inflammatory Parameters		
CRP (mg/L)	0.047 (0.015/0.080)	0.005
Fibrinogen (ng/ml)	0.022 (-0.006/0.050)	0.118
Albumin (g/dl)	-0.003 (-0.010/0.005)	0.464
Endocrine Parameters		
HOMA-IR*	0.02 (-0.01/0.05)	0.198
Leptin (ng/ml)	0.001 (-0.00003/0.003)	0.055
Adiponectin (ng/ml)	-0.01 (-0.016/-0.005)	<0.001
Insulin (mU/L)	0.007 (-0.0003/0.016)	0.060
C-Peptide (pM)*	-0.00001 (-0.0001/0.0001)	0.822
Cortisol (ng/ml)*	0.0003 (0.0002/0.0006)	<0.001

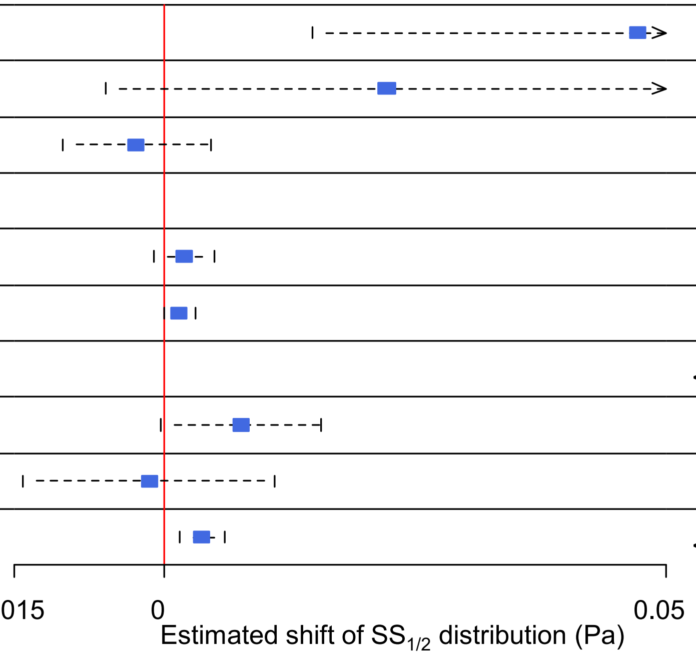

Supplementary Figure 3: Comparative Reference for Figures 3 & 4

A Association between RBCD and hemoglobin-oxygen dissociation (p50) in diabetes and nondiabetic groups stratified by RBCD ($SS_{1/2}$) tertiles. *(Data published in CVD 2024)*

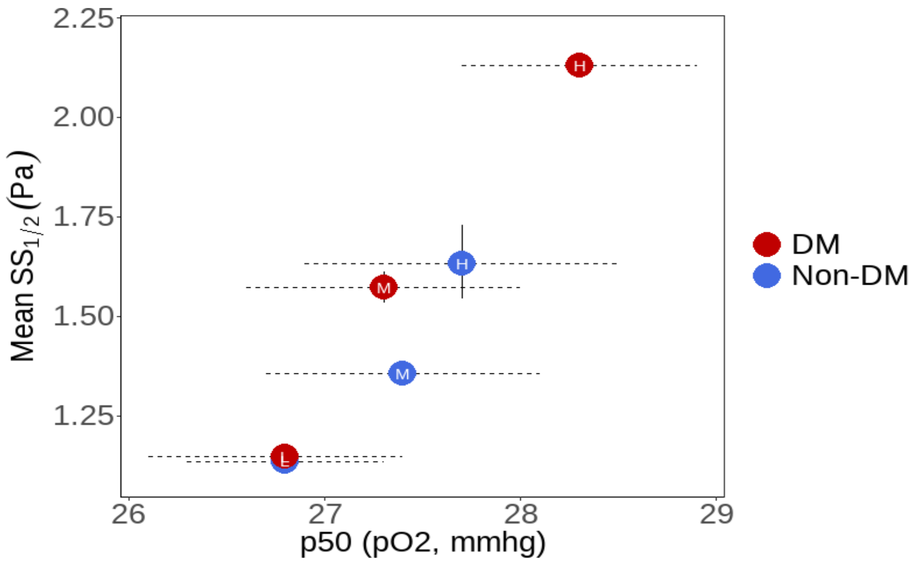

B Association between RBCD and osmotic fragility (Omin) diabetes and nondiabetic groups stratified by RBCD ($SS_{1/2}$) tertiles. *(Data published in CVD 2024)*

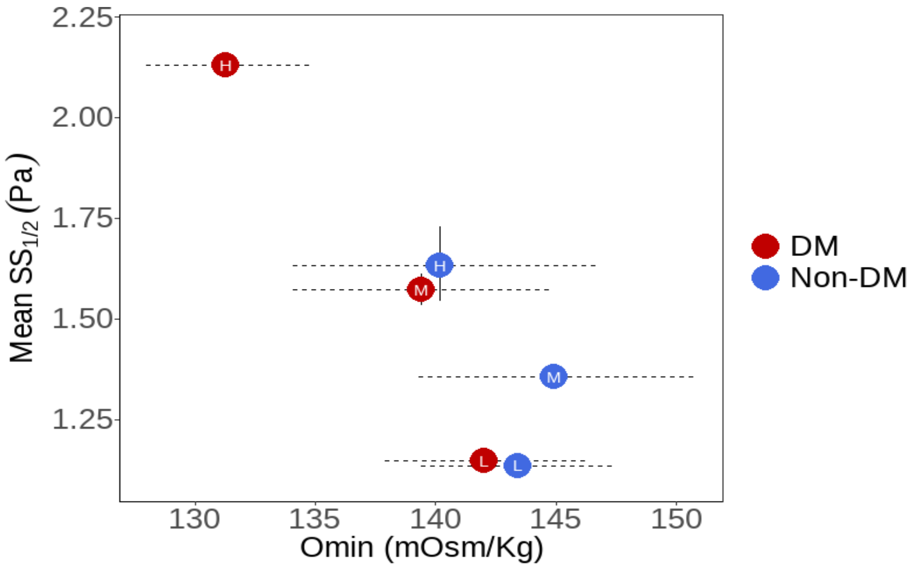

Supplementary Figure 4: Comparative Reference for Figure 4B

A

AI vs Osmotic fragility in participants with AI <75 and ≥75, on Box-Cox transformed scales.

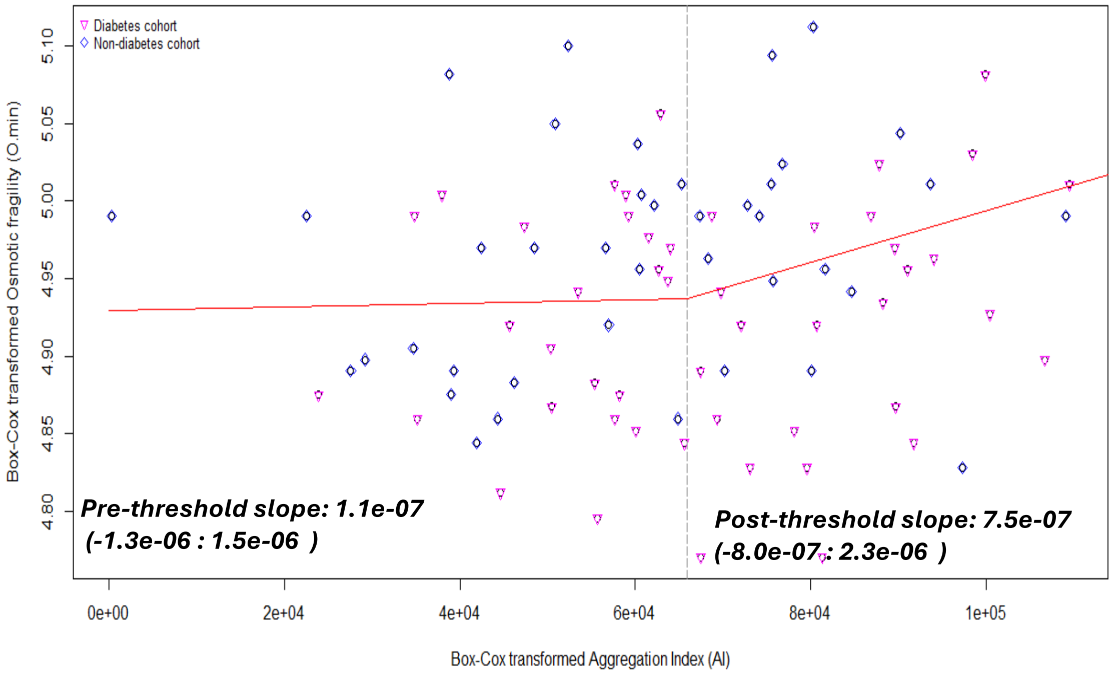

B

AI vs RBCD in participants with AI <75 and ≥75, on Box-Cox transformed scales.

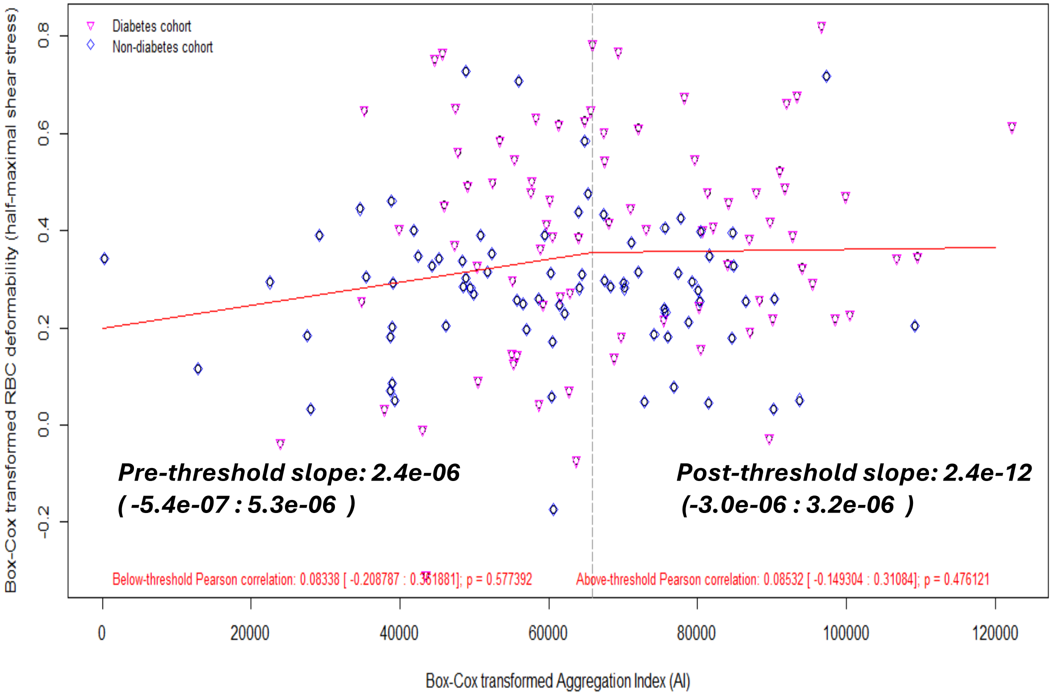

Supplemental Table 1: Values for AI tertile plots (Figures 3 and 4)

	Diabetes Cohort			Nondiabetic Controls		
**Covariates (mean/SD)**		**AI tertiles**			**AI tertiles**	
	**Low**	**Medium**	**High**	**Low**	**Medium**	**High**
Aggregation Index (AI)	66.6 (4.6)	75.1 (2.5)	84.2 (3)	59.6 (11.6)	72.9 (2.5)	80.7 (2.9)
RBCD ($SS_{1/2}$)	1.6 (0.6)	1.7 (0.5)	1.7 (0.5)	1.4 (0.3)	1.5 (0.3)	1.4 (0.3)
Osmotic fragility (Omin)	135.4 (9.3)	136.8 (10.6)	141.4 (10.8)	138.3 (9.7)	145.9 (9.6)	147.1 (10.8)
Hgb-oxygen dissociation (p50)	27.7 (1.9)	27.8 (1.2)	26.8 (1.6)	27 (1.8)	27.7 (2)	27.2 (1.6)
