# Supplementary material for: Altered RBC aggregability in diabetes: a threshold for pathophysiological structure-function RBC changes
Source: Cardiovasc Diabetol Endocrinol Rep. 2025 Dec 18;11:44. doi: 10.1186/s40842-025-00256-2 (PMC12713270; doi:10.1186/s40842-025-00256-2)
Supplement: Supplementary file 2 — Supplementary Material 2 [file 40842_2025_256_MOESM2_ESM.pdf]

SUPPLEMENTARY METHODS

In this supplementary methods section, we expand on the statistical approach used to estimate the AI Threshold—the qualitative switch in the directionality of association between the RBC Aggregation Index (AI) and RBC functional measure: hemoglobin-oxygen dissociation (p50). As explained in our prior work on RBCD[1], we utilized tertiles as finite-sample-driven compromise in how to explore subset-level changes in association without engendering the potential to be underpowered, especially as we have to account for diabetes status (the key factor for enrolling participants in our cross-sectional study cohorts. The AI-p50 association in subgroups stratified by AI-tertiles (Figure 3A in the main manuscript), entails a set of back-transformed values, taking the observed values alongside the predicted values from the Box-Cox transformed scales to their respective scales.

Analysis of cohort-specific AI-p50 associations (Figure S1 below), each indicated a distinct direction of association (via 'over'-smoothed regression lines of best fit) while simultaneously conveying (via more flexibly smooth estimates) a range of values, across which the direction of association might evolve to a qualitatively-distinct magnitude, from positive to negative association, as seen within both the diabetes and non-diabetic cohorts (Figure S1, below). While similar estimated changepoint (threshold) values were observed in the diabetes and control groups, the range of "ambiguous association" was much narrower in the control group vs the diabetes cohort (Figure S1). Notably, alternate cross-sectional study designs may well yield distinct AI threshold values due to distinct population mixtures of diabetes and non-diabetes persons.

We thus statistically estimated a possible AI threshold in the combined cohorts, along the range of observed AI values following the approach of Toms and Lesperance[2], resulting in the findings in the main manuscript, as portrayed in Figure 3B of the main manuscript. For the sake of appealing to all the assumptions inherent to regression modeling, the bivariate p50-AI relationship was explored with each measure being Box-Cox transformed, conditional on diabetes status as done within cohort plots (Figures S2). This approach involved adopting a piecewise-linear model for the hemoglobin-oxygen dissociation measure (p50) regressed upon AI, such that that linear association measure (slope) is allowed to change with a continuous transition in predicted outcome (transformed p50) values—at a point that is unknown--yet driven by the data (via maximum likelihood estimation). Due to modestly sized cohorts, we employed bootstrap estimation rather than relying on large-sample confidence intervals for this (diabetes- and p50-conditional Box-Cox transformed AI) transition point (AI threshold), as well as not

attempting to estimate unknown parameters that might characterize a less abrupt or 'sharp' transition (extending from 'Model 1' as labeled by Toms and Lesperance[2]).

Future studies of this qualitative transition from positive association to negative association, should they entail larger cohort sizes, would be better suited to a 'smoother' transition around this apparent changepoint threshold of AI, perhaps extending into multivariate regression that simultaneously explores potentially more subtle transitions along other RBC-, endocrine-, or inflammatory marker outcomes.

Figure S1: Cohort-stratified exploratory plots of bivariate transformed association between RBCA (AI) and RBC function (Hgb-O_2 dissociation, measured by p50); dotted-line polygons indicate each AI range of ambiguous association.

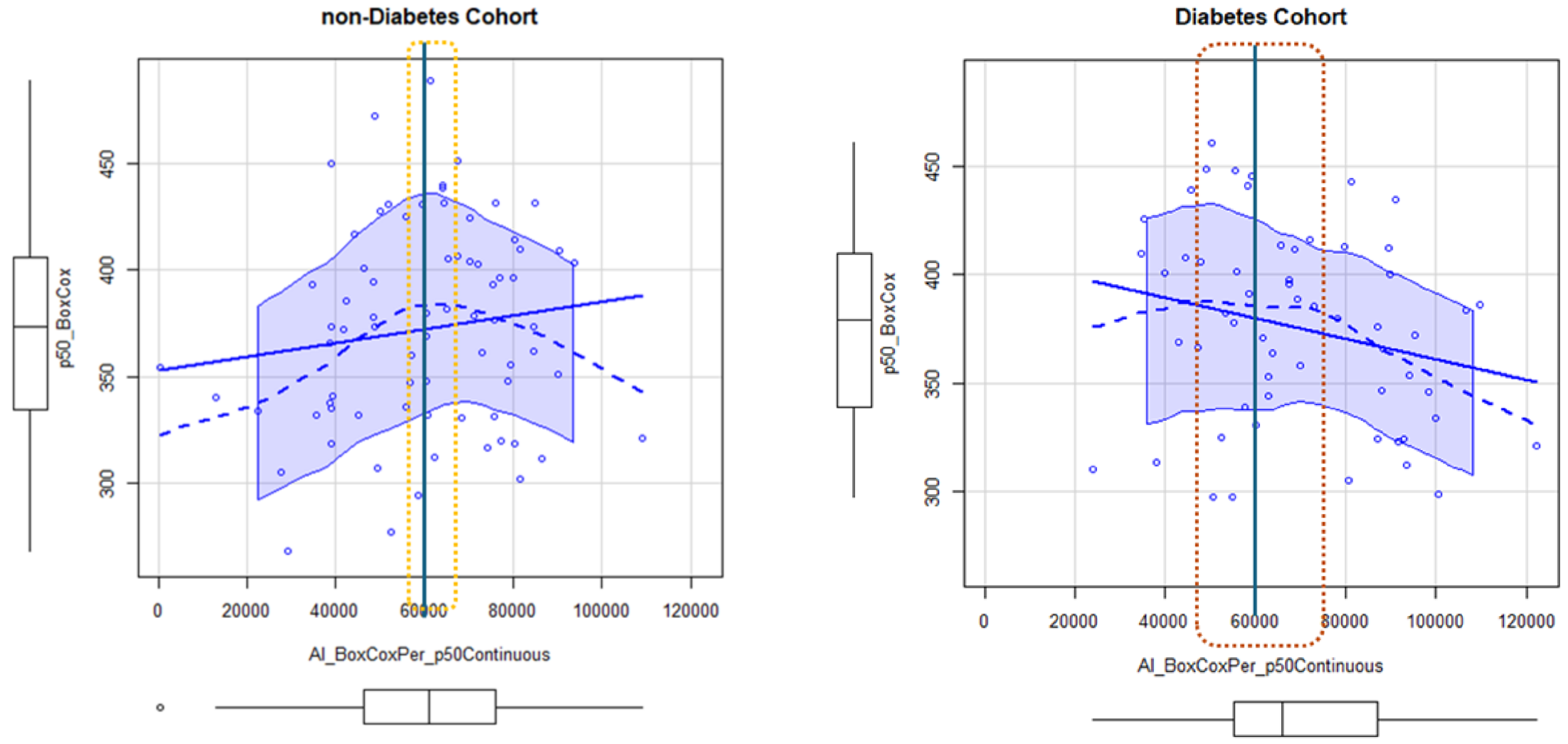

Figure S2: The bivariate Box-Cox transformed scatterplots and changepoint-analysis piecewise regression fits

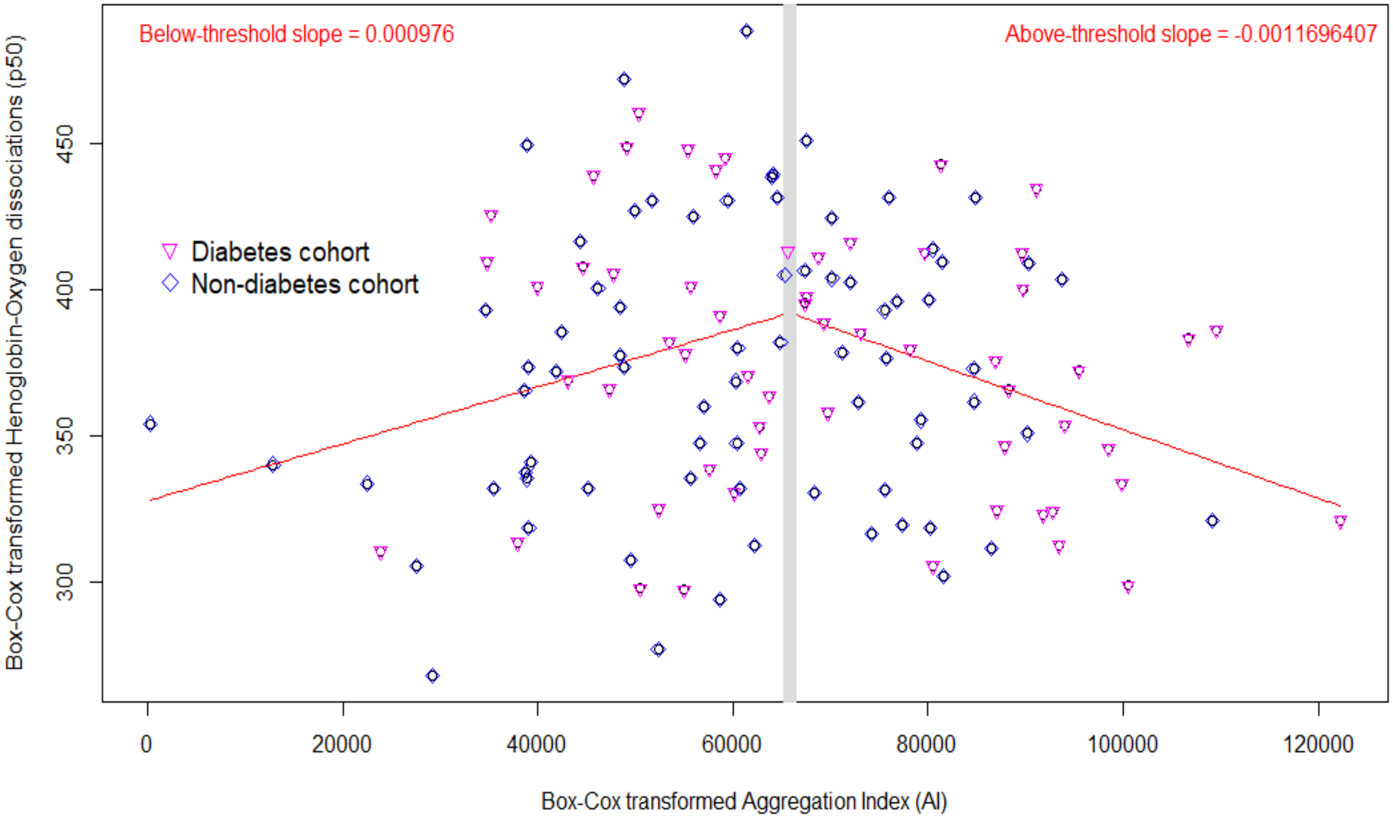

REFERENCES

1. Ebenuwa I, Violet PC, Tu H, et al. Altered RBC deformability in diabetes: clinical characteristics and RBC pathophysiology. Cardiovasc Diabetol 2024;23(1):370. (In eng). DOI: 10.1186/s12933-024-02453-2.
2. Toms JD, Lesperance ML. PIECEWISE REGRESSION: A TOOL FOR IDENTIFYING ECOLOGICAL THRESHOLDS. Ecology 2003;84(8):2034-2041. DOI: https://doi.org/10.1890/02-0472.
